# Causal relationship between plasma homocysteine levels and colorectal cancer: A Mendelian randomization study

**DOI:** 10.1097/MD.0000000000045773

**Published:** 2025-11-14

**Authors:** Xiaosong Ru, Linjun Wu, Yong Guo, Yinfeng Shen

**Affiliations:** a Department of Traditional Chinese Medicine, Hebei General Hospital, Shijiazhuang, China; b Hubei University of Chinese Medicine, Wuhan, China; c Department of Medical Oncology, The First Affiliated Hospital of Zhejiang Chinese Medical University, Hangzhou, China; d Hubei Provincial Hospital of Traditional Chinese Medicine Affiliated to Hubei University of Chinese Medicine, Wuhan, China; e Hubei Key Laboratory of Theory and Application Research of Liver and Kidney in Traditional Chinese Medicine, Wuhan, China.

**Keywords:** causality, colorectal cancer, homocysteine, Mendelian randomization

## Abstract

Given that homocysteine (Hey) may be a viable target for intervention and that there is uncertainty regarding the causal relationship between plasma Hcy levels and colorectal cancer (CRC), this study used Mendelian randomization (MR) to investigate the relationship between Hey and CRC. We summarized the data in this work using genome-wide association studies, identified single nucleotide polymorphisms that were strongly correlated with plasma Hcy levels as instrumental variables, and ran MR analysis on 2 separate sets of outcome data. To make sure the results were stable, a meta-analysis was carried out. MR-Egger, weighted median MR analysis, and the inverse variance method were among the specific analysis techniques used. The leave-one-out method, MR-Egger intercept, MR-PRESSO, and Cochran Q test were also used to assess the stability and dependability of the MR analysis results. In 2 separate European population-based datasets (UK Biobank: OR = 0.9992, 95% CI = 0.9963–1.0021, *P* = .5951, FinnGen: OR = 0.9771, 95% CI = 0.8370–1.1408, *P* = .7698), inverse variance method analysis did not reveal any significant causal connection between plasma Hcy levels and CRC. The MR-Egger and weighted median analyses yielded nonsignificant relationships. Both the Cochran Q test and the MR-Egger intercept indicated the absence of considerable heterogeneity and horizontal pleiotropy. The conclusions were further corroborated by the results of the MR-PRESSO analysis and leave-one-out. The pooled results of the Meta analysis also failed to demonstrate significant causality (OR = 0.9992, 95% CI = 0.9963–1.0021, *P* = .5914), therefore providing additional confirmation of the findings from the individual studies. There was no discernible causal relationship between plasma Hcy levels and CRC risk, according to MR analysis. The findings of this study indicate that while Hcy is a possible target for intervention, there may not be a direct causal relationship between it and the risk of CRC. This finding requires further validation in larger sample sizes and in different populations.

## 1. Introduction

Colorectal cancer (CRC) is a significant obstacle to global public health. It ranks as the third most frequently diagnosed malignant disease and the second greatest contributor to cancer-related fatalities on a global scale.^[1]^ CRC incidence and mortality have been increasing over the past decades, and understanding its etiology and underlying mechanisms is critical to devising effective prevention and treatment approaches. Environmental and genetic variables work together to impact the development of colorectal cancer (CRC). Dietary aspects, especially the intake of certain nutrients and micronutrients, have been implicated in the development of CRC.^[2]^ Therefore, the search for intervenable factors that are highly associated with colorectal carcinogenesis is clinically important in reducing the incidence of CRC.

Homocysteine (Hcy) is a sulfurous amino acid that originates from the metabolic process involving essential amino acids.^[3]^ Elevated plasma Hcy is considered to be toxic to cells and has been linked to many health problems. An elevated risk of developing a number of diseases, including cardiovascular disease, neurological disorders, diabetes, renal insufficiency, vitiligo, and cancer, has been demonstrated by elevated plasma Hcy levels.^[3–5]^ Nevertheless, the results of numerous studies that have examined the correlation between plasma Hcy levels and CRC have been inconsistent. While some studies have discovered that elevated plasma Hcy levels correspond to a higher likelihood of CRC,^[6–8]^ others have not found such a relationship.^[9–11]^ These research efforts are vulnerable to confounding variables and reverse causation, which might obscure the actual connection between Hcy and CRC.

Mendelian randomization (MR) studies are a valuable methodological tool for precisely evaluating the causal association among plasma Hcy and CRC. MR is the process of determining causal links by using variation in genomes as an instrumental variable.^[12]^ The main concept of MR relies on an arbitrary allocation of alleles during fertilization, which reduces confounding and offers a singular chance to clarify the causal link between exposure and outcome.^[13]^ To better understand whether adjusting Hcy levels could effectively reduce the risk of CRC, we conducted a 2-sample MR analysis to evaluate the causative connection between plasma Hcy and CRC.

## 2. Materials and methods

### 2.1. Study design

**Figure 1.**
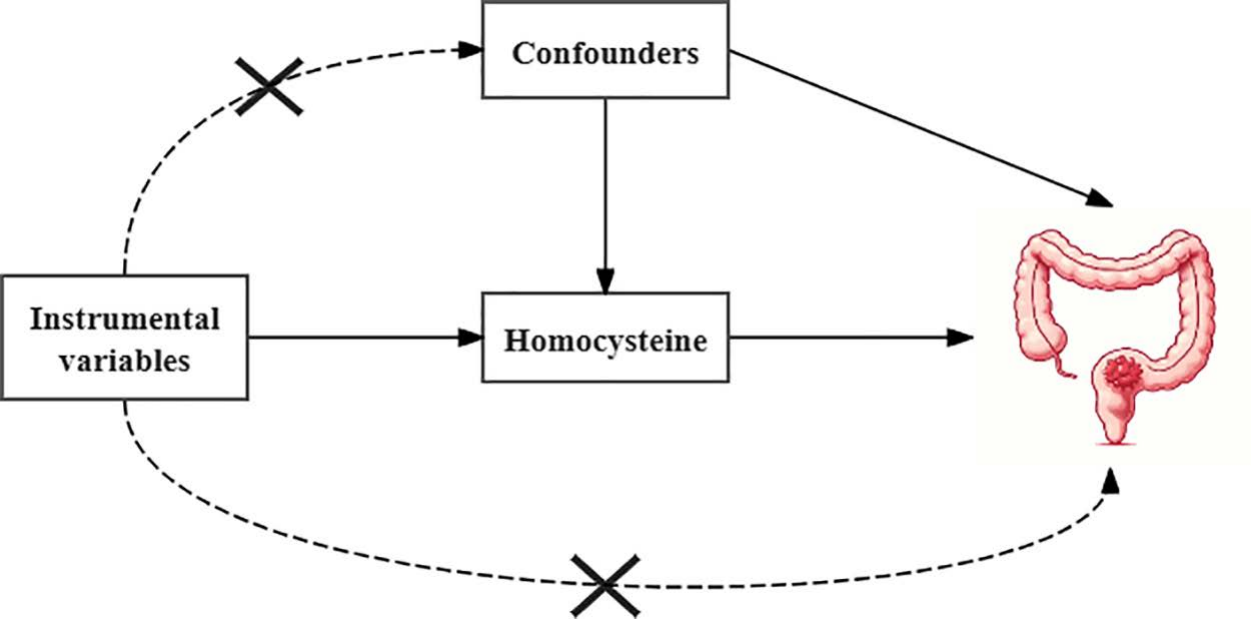
Mendelian randomization core assumption.

In this study, we utilized plasma Hcy as the exposure variable. Single nucleotide polymorphisms (SNPs) that showed a significant association with plasma Hcy were chosen as instrumental variables. CRC was designated as the outcome variable. We analyzed the causal link between plasma Hcy and CRC by employing MR methods. MR analyses must satisfy 3 core assumptions^[14]^: instrumental variables require a high degree of association with exposure; the effect of instrumental variables on the outcome must occur exclusively through the exposure, with no direct impact on the outcome; and instrumental variables should not be linked to the outcome via any confounding factors (Fig. 1). These 3 major assumptions form the cornerstone of MR analysis, and any violation of these assumptions may lead to biased or misleading results. Therefore, when performing an MR analysis, the fulfillment of these assumptions must be carefully considered and verified to ensure the validity and reliability of the analysis.

### 2.2. Data sources

The plasma Hcy data were acquired from a meta-analysis of genome-wide association studies (GWAS), which encompassed 10 distinct cohorts consisting of 44,147 people of European descent.^[15]^ The CRC data were extracted from the IEU database, which includes 377,673 persons of European descent. Among them, there were 5657 cases and 372,016 non-cases. In addition, CRC data from FinnGen data (version R10) containing 6847 cases and 314,193 controls were used for MR analysis in this study.^[16]^ Table 1 presents comprehensive information on the GWAS data.

**Table 1:** GWAS data detail information.

Exposure/Outcome	Data sources	Sample size	Ncase	Ncontrol	Population
Homocysteine	Joyce B J van Meurs et al	44,147	/	/	European
Colorectal cancer	UK Biobank	377,673	5657	372,016	European
Colorectal cancer	FinnGen	321,040	6847	314,193	European

### 2.3. Instrumental variable screening

To establish a robust relationship between instrumental variables and plasma Hcy, we implemented a comprehensive process of screening and evaluation. First, we selected SNPs that showed a significant association with the exposure from this GWAS meta-study. These SNPs were chosen as potential instrumental variables, meeting the criterion of *P* < 5 × 10^–8^. Afterward, to eliminate the potential interference of Linkage Disequilibrium, the independence of the selected instrumental variables was ensured by setting the parameters *r*^2^ = 0.001 and kb = 10,000. To further assess the applicability of the chosen SNPs, we utilized PhenoScanner V2 (http://www.phenoscanner.medschl.cam.ac.uk/) to investigate if these SNPs are associated with established confounding factors.^[17]^ Moreover, weak instrumental variables can affect the analysis results. Therefore, we evaluated the strength of these variables using the F-statistic. SNPs with an F-statistic below 10 were removed from the list of instrumental variables to prevent weak instrument bias.^[18]^ The formula was calculated as: F = β^2^/se^2^.

### 2.4. Data analysis

The core analytical method employed in this study was the inverse variance-weighted (IVW) approach. This method fundamentally involves calculating a weighted mean of the causal impact estimates for every instrumental variable, thereby synthesizing the overall causal effect estimates.^[19]^ For the purpose of enhancing the durability and dependability of the findings, the MR-Egger and weighted median techniques were employed as supplementary analytical approaches. To tackle potential heterogeneity and pleiotropy in instrumental variables, we conducted an in-depth analysis using the Cochran Q test and the MR-Egger intercept. The identification of instrumental variables that may lead to inconsistent effect estimates is facilitated by the Cochran Q test,^[20]^ while the MR-Egger intercept assesses, by calculating the intercept term that can be obtained after linear regression analysis, whether the presence of horizontal pleiotropy.^[21]^ Additionally, we introduced the MR-PRESSO method, which aims to identify and exclude outlier instrumental variables that may distort the overall causality estimation in order to perform a more accurate causality reestimation of the data set.^[22]^ Finally, each SNP was sequentially removed and reanalyzed using leave-one-out analysis to detect any notable influence of specific SNPs in the whole analysis results.^[23]^

### 2.5. Validation queue and meta-analysis

We assessed the reliability of the results by using the FinnGen database as a validation cohort and performing a meta-analysis of the IVW method on the results from both datasets. The statistical analyses in this study were conducted using R (version 4.3.2) and 2 R packages: “Twosample MR” (version 0.6.2) and “MRPRESSO” (version 1.0).

## 3. Results

### 3.1. Preliminary analysis results

From the GWAS Meta study, 18 SNPs were screened for significant association with plasma Hcy levels (*P* < 10^–8^). Five of these SNPs were excluded due to linkage disequilibrium. In the end, 13 SNPs were selected as instrumental variables for MR analysis. Their F-statistics were all above 10, indicating that the instrumental variables were statistically robust enough to minimize the possibility of bias resulting from weak instrumental variables.

**Figure 2.**
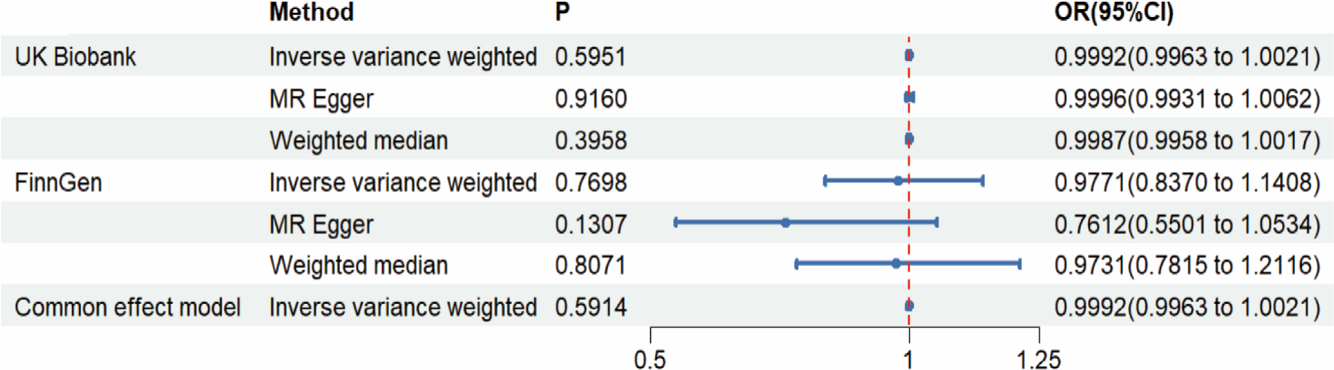
MR analysis results. MR = Mendelian randomization.

**Figure 3.**
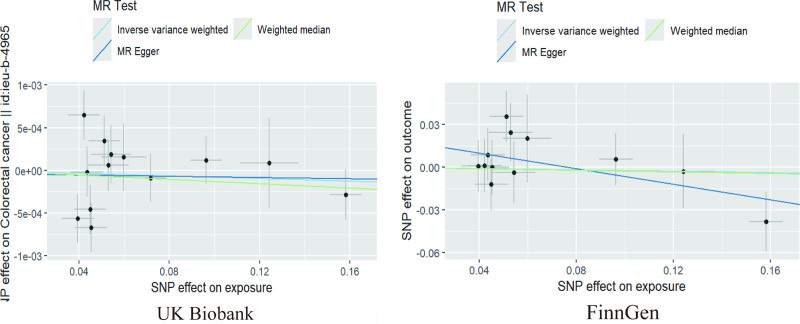
Scatterplot of MR analysis of 2 GWAS databases. GWAS = genome-wide association studies, MR = Mendelian randomization.

**Figure 4.**
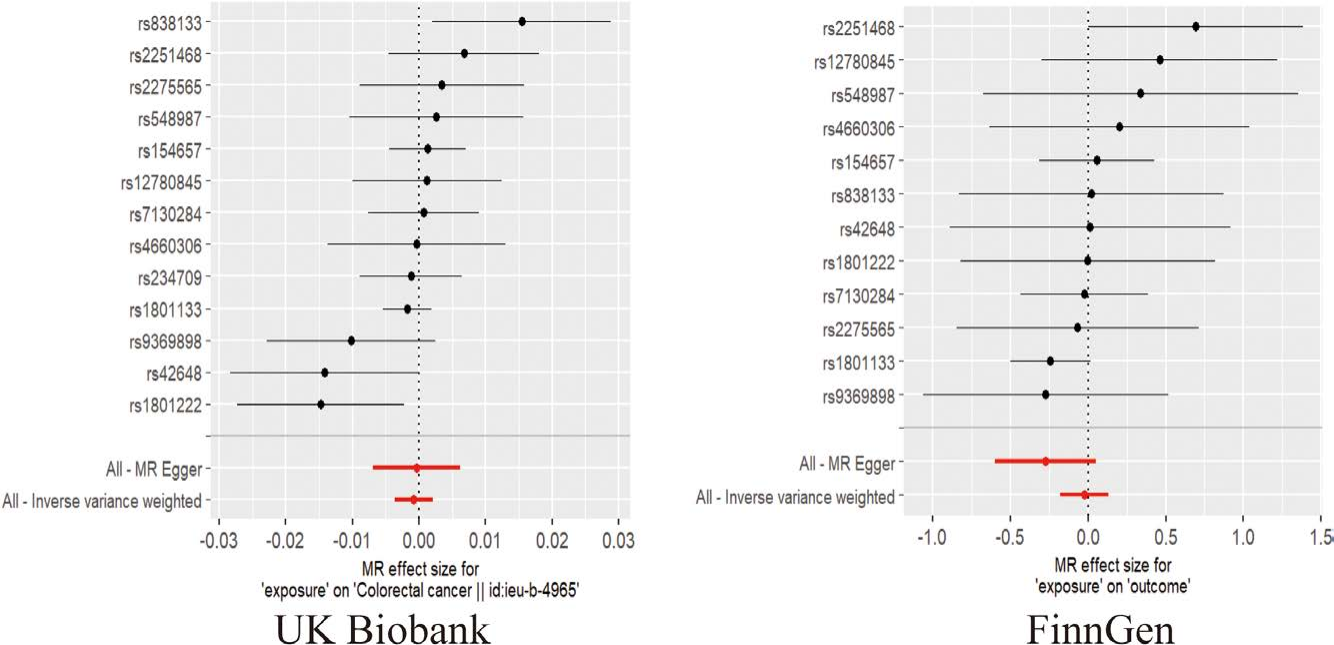
Forest plot for MR analysis of 2 GWAS databases. GWAS = genome-wide association studies, MR = Mendelian randomization.

**Figure 5.**
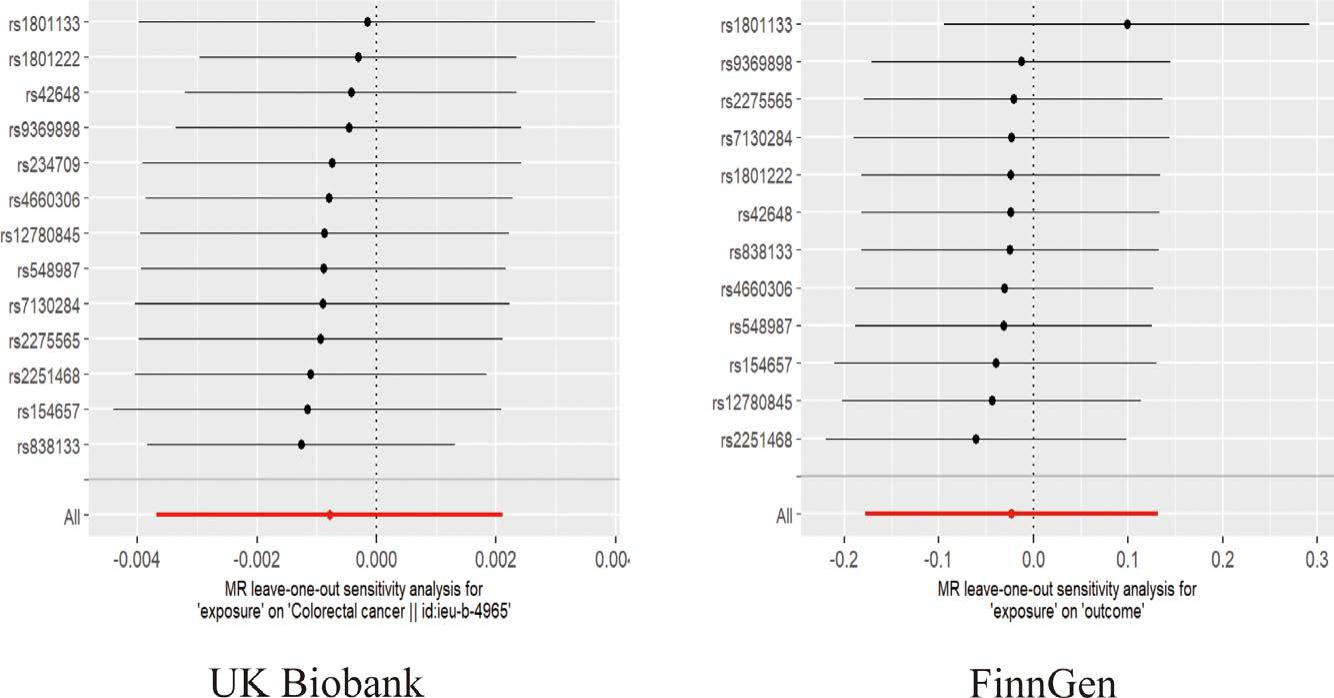
Results of leave-one-out method for 2 GWAS data. GWAS = genome-wide association studies.

The findings from the IVW analysis indicated that there was no statistically significant causal connection between plasma Hcy and CRC (OR = 0.9992, 95% CI = 0.9963–1.0021, *P* = .5951). In addition, this result was confirmed by MR-Egger (OR = 0.9996, 95% CI = 0.9931–1.0062, *P* = .9160) and the weighted median (OR = 0.9987, 95% CI = 0.9958–1.0017, *P* = .3958), both of which showed no significant causative. The findings are displayed in Figures 2–4. It was found that no heterogeneity among the instrumental variables included in the analysis, according to the IVW (*P* = .0785) and MR-Egger (*P* = .0542) results by Cochran Q test (Table 2). The horizontal pleiotropy did not affect the MR analysis results, as indicated by the MR-Egger intercept (Egger intercept = −0.000035, *P* = .8881). In addition, the MR-PRESSO analysis yielded not 1 significant outlier (*P* = .1340), enhancing the confidence of the results. The robustness of the MR analysis results was confirmed through a leave-one-out analysis. This analysis revealed that no individual instrumental variable had a significant impact on the causality estimations, thereby ensuring the reliability of the findings (Fig. 5).

**Table 2:** Sensitivity analysis results.

Exposure	Outcome	Cochran Q test		MR-Egger intercept test		MR-PRESSO global test
		IVW	MR-Egger	Intercept	*P*	*P*
Homocysteine	Colorectal cancer	0.0785	0.0542	−0.000035	.8881	.1340
Homocysteine	Colorectal cancer	0.5517	0.7418	0.0207	.1173	.3960

### 3.2. Validation of pairwise columns and meta-analysis results

As a validation cohort, we utilized the GWAS summary data from the FinnGen database to ensure the validity of the analytic findings. A total of 12 SNPs were acquired for MR analysis using this database. The analysis findings indicated no causal association between Hcy and CRC, as determined by the IVW (OR = 0.9771, 95% CI = 0.8370–1.1408, *P* = .7698), MR-Egger (OR = 0.7612, 95% CI = 0.5501–1.0534, *P* = .1307), and weighted median (OR = 0.9734, 95% CI = 0.7815–1.2116, *P* = .8071) methods (Figs. 2–4). In the validation cohort, both the Cochran Q test and the MR-Egger intercept did not indicate the presence of heterogeneity and pleiotropy (Table 2). In addition, MR-PRESSO (Table 2) and the leave-one-out method (Fig. 5) further confirmed the robustness of the findings.

After that, we carried out a meta-analysis of the IVW method. The meta-analysis revealed no heterogeneity (*I*² = 0.0%, H = 1.00, *P* = .7774), and a fixed-effects model was employed. The results (OR = 0.9992, 95% CI = 0.9963–1.0021, *P* = .5914) provided additional evidence to confirm the previous findings that there was no discernible causal link between Hcy and CRC (Fig. 2).

## 4. Discussion

Based on the publicly available GWAS database, this study employed a 2-sample MR to explore the causal link between plasma Hcy and CRC. The MR analysis of GWAS data from both cohorts, along with a meta-analysis of the results, indicated that a causal relationship between plasma Hcy levels and the development of CRC is unlikely.

Most of the causal findings regarding Hcy and CRC are based on previous observational studies, which have inherent limitations and are prone to bias. A meta-analysis revealed that elevated levels of Hcy were linked to a higher risk of CRC.^[8]^ And in a nested case-control study, serum Hcy was not found to be associated with the risk of CRC.^[10]^ Regarding the potential mechanism of the induction of cancer by Hcy, it may be closely related to factors such as metabolic disorders, oxidative stress, and inflammatory responses. Hcy is an intermediate amino acid in the metabolism of methionine and folic acid, which are essential for DNA methylation and synthesis. Disruption in Hcy metabolism can lead to DNA hypomethylation, abnormal DNA integration and repair, and chromosomal instability, which can subsequently affect the expression of tumor suppressor genes and proto-oncogenes, thereby promoting tumorigenesis.^[24,25]^ Excess oxygen radicals generated during the oxidation of Hcy in hyperhomocysteinemia may cause endothelial cell damage and DNA damage, which, while enhancing oxidative stress, may lead to mutations in key genes, such as p53 and ras, through endogenous attack on DNA, thereby increasing the risk of cancer development.^[26]^ At the same time, oxidative stress induced by hyperhomocysteinemia can activate multiple key signaling pathways, further inducing the expression of pro-inflammatory cytokines, chemokines, and cell adhesion molecules, thereby shaping a chronic inflammatory microenvironment.^[27]^ This persistent inflammatory state is closely associated with the initiation and progression of colorectal tumors. This study demonstrated that there is no direct association between plasma Hcy and CRC by means of MR analysis, suggesting that plasma Hcy is not a major driver of CRC etiology. In another MR analysis of Hcy and hormone-related cancers, it was hypothesized that Hey may not be an independent risk factor for cancer development, and that it acts through interactions with other exposure factors.^[28]^ In addition, colorectal carcinogenesis involves a multifactorial and multistep oncogenic process, including genetic susceptibility, environmental factors, dietary habits, and intestinal microbial communities.^[29]^ In contrast, the role of Hcy in this complex network may be nondeterministic, which further emphasizes the complexity of the role of Hcy in carcinogenesis.

The strength of this study lies in the use of MR analysis to effectively minimize the interference of confounding factors and reverse causation, thereby enhancing the reliability of the identified causal relationships. Additionally, multiple sensitivity analyses did not indicate any heterogeneity and pleiotropy, supporting the robustness of our findings. However, our study has certain limitations. First, all GWAS data used in this study were derived from populations of European ancestry, which may limit the generalizability of our findings to other ethnicities or populations. Therefore, these conclusions need to be further validated in cohorts with different ancestral backgrounds. Second, the GWAS data related to Hcy used in this study are relatively outdated; future research could incorporate more recent genetic datasets to improve timeliness. Finally, despite using methods to avoid the effect of horizontal pleiotropy, the complexity and uncertainty of the biological functions of genetic variants may still impact the results.

## 5. Conclusions

In summary, the MR analysis results from this study indicate that there is no definitive causal relationship between plasma Hcy levels and CRC. To confirm our findings and gain a comprehensive understanding of CRC pathogenesis, further research is needed to investigate the potential association in the future.

## Author contributions

**Conceptualization:** Xiaosong Ru.

**Data curation:** Xiaosong Ru.

**Software:** Xiaosong Ru.

**Supervision:** Yong Guo, Yinfeng Shen.

**Writing – original draft:** Xiaosong Ru, Linjun Wu.

**Writing – review & editing:** Yong Guo, Yinfeng Shen.
